# The CD200R1 microglial inhibitory receptor as a therapeutic target in the MPTP model of Parkinson’s disease

**DOI:** 10.1186/s12974-021-02132-z

**Published:** 2021-04-06

**Authors:** Neus Rabaneda-Lombarte, Joan Serratosa, Jordi Bové, Miquel Vila, Josep Saura, Carme Solà

**Affiliations:** 1 Department of Cerebral Ischemia and Neurodegeneration, Institut d’Investigacions Biomèdiques de Barcelona-Consejo Superior de Investigaciones Científicas (CSIC), Institut d’Investigacions Biomèdiques August-Pi i Sunyer (IDIBAPS), Barcelona, Spain; 2 Biochemistry and Molecular Biology Unit, School of Medicine, University of Barcelona, IDIBAPS, Barcelona, Spain; 3 Vall d’Hebrón Research Institute-CIBERNED, Barcelona, Spain; 4 Catalan Institution for Research and Advanced Studies (ICREA), Barcelona, Spain

**Keywords:** MPTP, Parkinson’s disease, Glia, Microglia, Immune response, CD200-CD200R1 system, Neuroinflammation, CD200Fc, CD200 KO mice

## Abstract

**Background:**

It is suggested that neuroinflammation, in which activated microglial cells play a relevant role, contributes to the development of Parkinson’s disease (PD). Consequently, the modulation of microglial activation is a potential therapeutic target to be taken into account to act against the dopaminergic neurodegeneration occurring in this neurological disorder. Several soluble and membrane-associated inhibitory mechanisms contribute to maintaining microglial cells in a quiescent/surveillant phenotype in physiological conditions. However, the presence of activated microglial cells in the brain in PD patients suggests that these mechanisms have been somehow overloaded. We focused our interest on one of the membrane-associated mechanisms, the CD200-CD200R1 ligand-receptor pair.

**Methods:**

The acute MPTP experimental mouse model of PD was used to study the temporal pattern of mRNA expression of CD200 and CD200R1 in the context of MPTP-induced dopaminergic neurodegeneration and neuroinflammation. Dopaminergic damage was assessed by tyrosine hydroxylase (TH) immunoreactivity, and neuroinflammation was evaluated by the mRNA expression of inflammatory markers and IBA1 and GFAP immunohistochemistry. The effect of the modulation of the CD200-CD200R1 system on MPTP-induced damage was determined by using a CD200R1 agonist or CD200 KO mice.

**Results:**

MPTP administration resulted in a progressive decrease in TH-positive fibres in the striatum and TH-positive neurons in the substantia nigra pars compacta, which were accompanied by transient astrogliosis, microgliosis and expression of pro- and anti-inflammatory markers. CD200 mRNA levels rapidly decreased in the ventral midbrain after MPTP treatment, while a transient decrease of CD200R1 mRNA expression was repeatedly observed in this brain area at earlier and later phases. By contrast, a transient increase in CD200R1 expression was observed in striatum. The administration of a CD200R1 agonist resulted in the inhibition of MPTP-induced dopaminergic neurodegeneration, while microglial cells showed signs of earlier activation in CD200-deficient mice.

**Conclusions:**

Collectively, these findings provide evidence for a correlation between CD200-CD200R1 alterations, glial activation and neuronal loss. CD200R1 stimulation reduces MPTP-induced loss of dopaminergic neurons, and CD200 deficiency results in earlier microglial activation, suggesting that the potentiation of CD200R1 signalling is a possible approach to controlling neuroinflammation and neuronal death in PD.

## Background

Neuroinflammation, in which activated glial cells are involved, is one of the hallmarks of Parkinson’s disease (PD) [80]. The presence of activated glia, mainly microglia, in the post-mortem brain in patients with PD was described many years ago using histological techniques [53]. More recently, imaging studies have also shown enhanced microglial activation in the brain of patients affected by this disorder, even at very early disease stages [20, 30]. The detection of inflammation markers in the brain, serum and cerebrospinal fluid in PD patients further supports the involvement of an inflammatory response in the pathological process [18, 57, 68]. Although the role of neuroinflammation in the development of the disease remains unclear, it is thought to be involved in the progression of the pathology and to be a potential target for novel therapeutic strategies [40–42, 49]. Recently, it has also been proposed that inflammation in the periphery and its posterior transmission to the brain may be one of the initial steps in the development of PD [27].

The inhibition of glial activation results in neuroprotection in both in vitro [28, 84] and in vivo [7, 34, 60] experimental models of PD. In addition, epidemiological studies show that chronic administration of anti-inflammatory agents reduces the risk of PD, although the results are still controversial [64]. However, no benefit has been observed so far in clinical trials testing anti-inflammatory agents in PD patients, suggesting that new targets should be found or that the window of action of these kinds of compounds may correspond to early/prodromal phases of the disease. Moreover, it has been suggested that modulation of glial activation would be a better therapeutic approach in neurodegenerative diseases than trying to block or prevent the inflammatory response of glial cells [58].

Glial cells, mainly microglia, are the predominant mediators of the innate immune response in the CNS [12, 44, 67]. In physiological conditions, glial cells are maintained in a quiescent/surveillant state by an inhibitory brain environment. However, they are ready to react to changes in the extracellular milieu in order to recover tissue homeostasis. Glial activation is a physiological response, but it can be associated with the production of potentially neurotoxic molecules. Consequently, glial activation has to be tightly regulated and resolved when it is no longer necessary [67]. The chronic presence of glial activation in PD suggests that these mechanisms of control have been overloaded.

The control of glial activation involves both contact-dependent and contact-independent mechanisms [72]. Several ligand-receptor pairs act as contact-dependent mechanisms, such as CX3CL1-CX3CR1, CD47-SIRPα, sialic acids (siglecs), or CD200-CD200R1. We focused our interest on the CD200-CD200R1 system [35, 52]. In the central nervous system, the CD200 ligand is mainly expressed by neurons, but also by astrocytes and oligodendrocytes, while CD200R1 is an inhibitory receptor expressed in microglial cells. CD200-CD200R1 interaction activates a signal transduction pathway that results in the inhibition of the expression of pro-inflammatory molecules. Alterations in the expression of CD200 and CD200R1 have been described in the brain in multiple sclerosis and Alzheimer’s disease patients [38, 39, 77]. In addition, polymorphisms in the promoter of the CD200R1 gene, which result in a reduced transcriptional activity of the promoter, have been associated with a higher risk of PD [46]. CD200 deficiency or treatment with a CD200R1-blocking antibody resulted in an enhanced pathology in an experimental model of multiple sclerosis, while the administration of a CD200R1 agonist resulted in an attenuated pathology [26, 55]. Moreover, increased neuronal damage was observed in an experimental model of PD (6-hydroxydopamine-lesioned rats) after the administration of a CD200R1-blocking antibody [87]. Alterations in the cerebral expression of CD200 and CD200R have been described in the LPS and the α-synuclein overexpression models of PD [79]. These observations suggest that the CD200-CD200R1 system may be a potential target for attenuating microglial activation and the resulting neurotoxicity.

In the present work, we studied the involvement of the CD200-CD200R1 system in the development of neurodegeneration in a mouse model of PD, the acute MPTP (1-methyl-4-phenyl-1,2,3,6-tetrahydropyridine) model. MPTP mouse models are among the most widely used models of PD [4]. They reproduce the naturally occurring neurodegeneration and are useful for studying dopaminergic neuron neurodegeneration, mitochondrial dysfunction and neuroinflammation [54]. We first characterized the temporal pattern of dopaminergic neuronal death, neuroinflammation and CD200 and CD200R1 expression after MPTP administration. Then, we evaluated the involvement of the CD200-CD200R1 system using two different approaches: the potentiation of CD200R1 signalling with a CD200R1 agonist and the disruption of the CD200-CD200R1 function using CD200 knockout (KO) mice. Our results show that MPTP-induced neurodegeneration and neuroinflammation are accompanied by alterations in the expression of CD200 and CD200R1, which probably facilitates the development of a microglial pro-inflammatory phenotype. The stimulation of CD200R1 results in neuroprotection, pointing to CD200R1 as an interesting target to act against neuroinflammation and the resulting neurotoxicity in neurodegenerative disorders such as PD.

## Methods

Experiments were carried out in accordance with European Union directives (2010/63/EU) and Spanish regulations (Real Decreto 1386/2018) on the use of laboratory animals and were approved by the Ethics and Scientific Committees of Barcelona University and the *Hospital Clínic Provincial de Barcelona*, CSIC and Vall d’Hebrón Research Institute Animal Ethical Experimental Committee.

### Animals

C57BL/6N mice were obtained from Charles River (Lyon, France). CD200-deficient mice (CD200 −/−) were kindly provided by Dr. David Copland (University of Bristol, United Kingdom). The CD200 −/− mice had been generated at DNAX (Palo Alto, CA) [26] using C57BL/6 embryonic stem cells. Although C57BL/6 mice are usually used in the MPTP model, increased toxicity of MPTP is observed in certain substrains such as C57BL/6 J mice [31, 54]. We submitted the CD200 −/− mice to background strain characterization (Jackson Laboratories), and they were identified as 67% C57BL/6 J and 33% C57BL/6N. We generated a colony by crossing CD200 −/− mice with CD200 +/+ C57BL/6N mice to yield an F1 offspring with a reduced C57BL/6 J background (33% C57BL/6 J and 67% C57BL/6N). Male and female heterozygous mice (CD200 +/−) of the F1 offspring were crossed to obtain the CD200 +/+ and CD200 −/− mice that were used thereafter. The genotype of the mice used in the experiments was corroborated by genotyping tail tissue.

### Treatments and tissue collection

Mice between 11 and 15 weeks old received MPTP injections following an acute regimen [31], which consisted of one intraperitoneal (i.p.) injection of MPTP-HCl (20 mg/kg or 18 mg/kg, Sigma-Aldrich, Madrid, Spain) every 2 h, reaching a total of 4 doses in 1 day. Control mice received saline injections. Mice were sacrificed at indicated time points following the last MPTP injection. Male mice were used in all the experiments, with the exception of the experiments with CD200-deficient mice, in which females were used in the control group because not enough male mice were available to complete all the experimental groups.

#### Time course experiment

Male C57BL/6N mice (eight mice per group) were intraperitoneally administered saline or 20 mg/kg MPTP, following the acute regimen and sacrificed at 2 h, and at 1, 2, 4 and 7 days after the last MPTP injection. Control mice were sacrificed at the last time point. Mice were anaesthetized with sodium pentobarbital (5%, i.p.) and perfused with physiological saline (0.9% NaCl). Brains were removed and the two hemispheres were separated down the longitudinal fissure. Right hemispheres were processed for immunohistochemistry and left hemispheres for gene expression analysis.

#### CD200Fc experiment

C57BL/6N mice were administered with 20 mg/kg MPTP, following the acute regimen. The CD200R1 agonist (CD200Fc; 1.8 mg/kg or 3.6 mg/kg, dissolved in phosphate-buffered saline, PBS) or its corresponding isotype control (1.8 mg/kg or 3.6 mg/kg, dissolved in PBS) was administered twice, by intraperitoneal injection, 30 min before MPTP injections and 24 h after the last MPTP injection. Six groups of eight mice were administered with (1) saline, (2) MPTP, (3) 1.8 mg/kg CD200Fc and MPTP, (4) 1.8 mg/kg isotype control and MPTP, (5) 3.6 mg/kg CD200Fc and MPTP, and (6) 3.6 mg/kg isotype control and MPTP. The CD200Fc and its corresponding isotype control were kindly provided by Genentech Inc. The CD200Fc is a fusion protein constituted by the extracellular domain of mouse CD200 and the mouse IgG2a Fc region. Mouse anti-ragweed IgG2a was used as isotype control. Animals were sacrificed at 7 days after the last MPTP injection. Mice were anaesthetized with sodium pentobarbital and perfused with physiological saline. The brains were processed for immunocytochemistry.

#### CD200 KO mice experiments

CD200 +/+ and CD200 −/− mice were administered with saline or 18 mg/kg MPTP following the acute regimen and sacrificed at 7 days after the last MPTP injection. In this case, the MPTP doses were reduced from 20 to 18 mg/kg because in a preliminary study we detected a higher MPTP sensitivity in mice of the CD200 colony than in the commercial mice used in the previous experiments (data not shown), probably due to their different genetic background. In a first experiment, four groups of mice were treated as follows: (1) control group of CD200 +/+ mice treated with saline (*n* = 8), (2) CD200 +/+ mice treated with MPTP (*n* = 12), (3) CD200 −/− mice treated with saline (*n* = 8), (4) CD200 −/− mice treated with MPTP (*n* = 11). Female mice were used in the saline groups because not enough male mice were available to complete all the experimental groups. Mice were sacrificed 7 days after the last administration of MPTP. In a second experiment, mice were sacrificed 1 day after the last MPTP injection. The same four experimental groups were used, with female mice (CD200 +/+ and CD200 −/−, *n* = 8 per group) again used for the saline controls, while male mice (CD200 +/+, *n* = 11 and CD200 −/−, *n* = 9) were injected with MPTP. Mice were anaesthetized with sodium pentobarbital and perfused with physiological saline, followed by ice-cold 4% paraformaldehyde (Panreac, Barcelona, Spain) diluted in 0.2 M PBS containing 0.15 M sodium phosphate dibasic (Sigma-Aldrich) and 0.05 M sodium phosphate monobasic (Sigma-Aldrich). Brains were processed for immunohistochemistry.

### Genotyping

To evaluate the genotype of mice (CD200 +/+, CD200 +/−, CD200 −/−), genomic DNA from tail tissue was extracted and amplified with REDExtract-N-Amp™ Tissue PCR Kit (Sigma-Aldrich) following the manufacturer’s instructions. The amplified DNA was loaded onto a 2% agarose gel, together with a DNA ladder (Thermo Fisher Scientific, Waltham, MA, USA). For DNA detection, Midori green nucleic acid staining solution was used (Nippon Genetics Europe, Dueren, Germany) and images were obtained using a UV Transilluminator (Gel Doc System, Bio-Rad Laboratories, Hercules, CA, USA). The primers (Integrated DNA Technology, IDT, Skokie, IL, USA) used are listed in Table 1. The two pairs of primers were used in each reaction because each DNA sample was screened for both the normal and the mutant allele by using a single PCR. DNA from CD200 +/+ mice was amplified by mCD200 primers, producing one band of 506 bp. DNA from CD200 +/− mice was amplified by mCD200 and mNEO primers, producing one band of 506 bp and one band of 596 bp. DNA from CD200 −/− mice was amplified by mNEO primers, producing one band of 596 bp.

**Table 1:** Primers used for genotyping

Genotype	Forward 5′ ➔ 3′	Reverse 5′ ➔ 3′	Amplicon
CD200 +/+	mCD200-Fw GAAGACAAACCTAGCGGAGACATTAC	mCD200-Rv CTCTTCAGCAATATCACGGGTAGC	506 bp
CD200 −/−	mNEO-Fw GGGAGTGGAACTGTAGAAGGGTG	mNEO-Rv AGGCTATTCGGCTATGACTGGG	596 bp

### Immunohistochemistry

Brains removed after the perfusion were immersed overnight in 4% paraformaldehyde diluted in 0.2 M PBS at 4 °C and then for 48 h in 30% sucrose (Scharlau, Barcelona, Spain) at 4 °C. Finally, brains were frozen on dry ice and kept at − 80 °C. Serial coronal brain sections (20 μm thick) containing the striatum or the substantia nigra (SN) were obtained with a cryostat (Leica CM1950, Nussloch, Germany) at − 23 °C, collected in 0.1 M PBS containing 0.01% sodium azide (Sigma-Aldrich) and stored at 4 °C until used.

Assessment of nigrostriatal integrity was performed in tyrosine hydroxylase (TH)-immunostained tissue sections. TH immunohistochemistry was performed in four representative sections of striatum per animal, covering different striatal levels and in every sixth section throughout the entire SN pars compacta (SNpc), yielding twelve serial sections per animal. TH immunoreactivity in striatum was evaluated by optical densitometry and the number of TH-positive neurons in the SNpc was estimated by stereology as explained in detail in the next section.

To assess glial reactivity in the striatum and SN, three to four representative sections of each area per animal were immunostained against GFAP, and three to four against IBA1. Changes in IBA1 and GFAP immunoreactivity in striatum and SNpc were qualitatively evaluated, taking into account the increase in the number of positive cells with hypertrophied morphology and/or intensified IBA1 or GFAP staining (+: few cells, ++: moderate number of cells, +++: many cells with hypertrophied morphology and/or intensified IBA1 or GFAP staining). In some experiments, quantification of IBA1 immunostaining was performed as explained in detail in the next section.

Free-floating fixed sections were first permeated in Tris-buffered saline (TBS) containing 10% methanol and 3% H_2_O_2_ for 5 min, rinsed in PBS and blocked with 5% normal goat serum (Vector Laboratories, Inc., Burlingame, CA, USA) for 1 h at room temperature. Sections were incubated overnight at 4 °C in gentle agitation with primary antibodies (Table 2) diluted in TBS containing 2% normal goat serum. After being rinsed in PBS, the sections were incubated for 1 h at room temperature with the corresponding secondary biotinylated antibodies (Table 2) diluted in TBS with 2% normal goat serum, followed by PBS washes. To amplify signal intensity, tissue sections were incubated with the avidin-biotin complex (ABC) Reagent (Thermo Fisher Scientific) for 30 min and washed with PBS. For the visualization of peroxidase activity, we used 3,3′-diaminobenzidine (Sigma-Aldrich). Sections were mounted on gelatin-coated slides and air dried overnight. TH-immunostained sections of the SN were counterstained with Nissl stain. Briefly, after 10 min of incubation with chloroform, sections were rehydrated through 95% then 70% ethanol to distilled water, stained in 0.1% cresyl violet solution for 10 min, rinsed quickly in distilled water and differentiated in acetic acid + 70% ethanol (2 drops of glacial acetic acid/100 mL of 70% ethanol). All sections were dehydrated through 70%, 95% and 100% ethanol to xylene and coverslips were placed over slides using DPX mounting medium (Sigma-Aldrich).

**Table 2:** Antibodies used in immunohistochemistry

Epitope	Source	Dilution	Reference	Company
**Primary antibodies**				
Anti-GFAP	Rabbit	1/2000	Z0334	Dako
Anti-IBA1	Rabbit	1/1000	019-19741	Wako
Anti-TH	Rabbit	1/5000 (St) 1/2000 (SN)	ab112	Abcam
**Biotinylated secondary antibodies**				
Goat IgG	Horse	1/1000	PI-9500	Vector
Rabbit IgG	Goat	1/1000	BA1000	Vector

*GFAP*, glial fibrillary acidic protein; *IBA1*, ionized calcium binding adaptor molecule 1; *SN*, substantia nigra; *St*, striatum; *TH*, tyrosine hydroxylase

### Quantitative morphology

#### Striatal TH-positive fibres

The extent of striatal dopaminergic denervation was measured by optical densitometry in four TH-immunostained sections from each animal, as previously described [59, 62]. Sections were scanned in an Epson Perfection V750 PRO scanner, and the grey intensity of the staining in the striatum of both hemispheres of each section was measured using SigmaScan Pro 5.0 software (Systat Software, USA). The measured values were corrected for non-specific background staining by subtracting values obtained from the cortex. The optical density (OD) was calculated with the formula OD = −log (intensity in the striatum / intensity in the cortex).

#### SNpc TH-positive neurons

The total number of TH-positive neurons in the SNpc was estimated by stereological quantification using twelve regularly spaced sections per animal and employing the optical fractionator principle with Stereoinvestigator Software (MBF Bioscience, Williston, VT) on a Zeiss ImagerD1 microscope, as previously described [45, 62]. The SNpc was delineated for each section and probes for stereological counting were applied to the map obtained (size of counting frame was 50 × 50 μm spaced by 250 × 250 μm). Only those TH-positive neurons with their nuclei included within the counting frame were counted.

#### SN IBA1-positive microglia

Microglial reactivity in SN was measured in four IBA1-immunostained sections from each animal. Images of SN of each section were obtained using a × 4 objective of an Olympus IX70 microscope (Olympus, Okoya, Japan) and a digital camera (CC-12, Olympus Soft Imaging Solutions GmbH, Hamburg, Germany). Three parameters were measured:

- IBA1-labelled area

A grey-level threshold was fixed for all the sections and the area occupied by IBA1 staining in the total SN and SNpc of each animal was measured using ImageJ 1.50 software (National Institutes of Health). Values were expressed as percentage of area occupied by IBA1 staining in SNpc or total SN.

(b) IBA1 optical density

The mean grey intensity in IBA1-stained cells in the total SN and SNpc of each section was measured using ImageJ 1.50 software (National Institutes of Health). Non-specific background staining was obtained from an IBA1 staining free area. The optical density (OD) was calculated with the formula OD = −log (intensity in SNpc or total SN / intensity of background area).

(iii) IBA1-positive cells

The number of IBA1-positive cells in the total SN and SNpc of each section was measured using ImageJ 1.50 software (National Institutes of Health).

### RNA extraction and quantitative real-time polymerase chain reaction (qRT-PCR)

Total RNA was extracted at the indicated time points from striatal and ventral midbrain samples using the Trizol method (Tri®Reagent, Sigma-Aldrich) following the manufacturer’s instructions. Total RNA concentration was measured on a Nanodrop 1000 spectrophotometer (Thermo Fischer Scientific). The resulting RNA was stored at − 80 °C until further use. One microgram of RNA was reverse transcribed with random and oligo(dT) primers by using qScriptTM cDNA Synthesis Kit (Quanta Biosciences, Beverly, CA, USA) according to the manufacturer’s instructions. The reverse transcription was performed using a thermal cycler under the following protocol: 25 °C for 5 min, 42 °C for 30 min and 85 °C for 5 min. The resulting cDNA was stored at − 20 °C until used. cDNA was diluted 1/30 and 5 ng of cDNA was used to perform qRT-PCR with SYBR Green Mix (PCR Biosystems, London, UK) in 15 μL of final volume, using the iCycler MyIQ apparatus (Bio-Rad Laboratories). Samples were run at 95 °C for 2 min to activate the polymerase followed by 40 cycles consisting of denaturation at 95 °C for 15 s, annealing at 60 °C for 30 s and extension at 72 °C for 15 s. The primers used (Integrated DNA Technology) are shown in Table 3. Relative gene expression values were calculated with the 2^−ΔΔCt^ method [50]. GAPDH and βActin were used as reference genes.

**Table 3:** Primers used for qRT-PCR

Species: ***Mus musculus***			
Target mRNA	Accession number	Forward primer (5′➔3′)	Reverse primer (5′➔3′)
Arg1	NM_007482.3	TTGCGAGACGTAGACCCTGG	CAAAGCTCAGGTGAATCGGC
Cd200full	NM_010818.3	GGGCATGGCAGCAGTAGCG	TGTGCAGCGCCTTTCTTTC
Cd200tr	NM_001358443.1	GATGGGCAGTCTGTGGAAGTG	GAGAACATCGTAAGGATGCAGTTG
Cd200R1	NM_021325.3	AGGAGGATGAAATGCAGCCTTA	TGCCTCCACCTTAGTCACAGTATC
COX2	NM_011198.4	TGCAGAATTGAAAGCCCTCT	CCCCAAAGATAGCATCTGGA
gp91phox	NM_007807.5	ACTCCTTGGGTCAGCACTGGCT	GCAACACGCACTGGAACCCCT
IL10	NM_010548.2	TGAATTCCCTGGGTGAGAAG	ACACCTTGGTCTTGGAGCTT
IL1β	NM_008361.4	TGGTGTGTGACGTTCCCATTA	CAGCACGAGGCTTTTTTGTTG
IL6	NM_031168.2	CCAGTTTGGTAGCATCCATC	CCGGAGAGGAGACTTCACAG
iNOS	NM_010927.3	GGCAGCCTGTGAGACCTTTG	GCATTGGAAGTGAAGCGTTTC
MR	NM_008625.2	TCTTTTACGAGAAGTTGGGGTCAG	ATCATTCCGTTCACCAGAGGG
Nrf2	NM_010902.4	GATCCGCCAGCTACTCCCAGGTTG	CAGGGCAAGCGACTCATGGTCATC
TGFβ	NM_011577.2	TGCGCTTGCAGAGATTAAAA	AGCCCTGTATTCCGTCTCCT
TNFα	NM_013693.3	TGATCCGCGACGTGGAA	ACCGCCTGGAGTTCTGGAA
**Reference genes**			
βactin	NM_007393.5	CAACGAGCGGTTCCGATG	GCCACAGGATTCCATACCCA
Gapdh	NM_008084.3	GGTGAAGGTCGGTGTGAACG	CTCGCTCCTGGAAGATGGTG

*Arg1*, arginase 1; *CD200full*, full-length CD200; *CD200tr*, truncated CD200; *CD200R1*, CD200 receptor 1; *COX2*, cyclooxygenase 2; *Gapdh*, glyceraldehyde-3-phosphate dehydrogenase; *gp91phox*, catalytic subunit of NADPH oxidase; *IL10*, interleukin 10; *IL1β*, interleukin 1β; *IL6*, interleukin 6; *iNOS*, inducible nitric oxide synthase; *MR*, mannose receptor; *Nrf2*, nuclear factor erythroid 2-related factor 2; *TGFβ*, transforming growth factor β; *TNFα*, tumour necrosis factor α

### Data presentation and statistical analysis

The results are presented as the mean + SEM values. Statistical analyses were performed using one-way analysis of variance (ANOVA) followed by the Newman-Keuls post hoc test and two-way ANOVA followed by the Bonferroni post hoc test. Values of *p* < 0.05 were considered statistically significant.

## Results

### Temporal pattern of dopaminergic damage in MPTP-treated mice

**Fig. 1:**
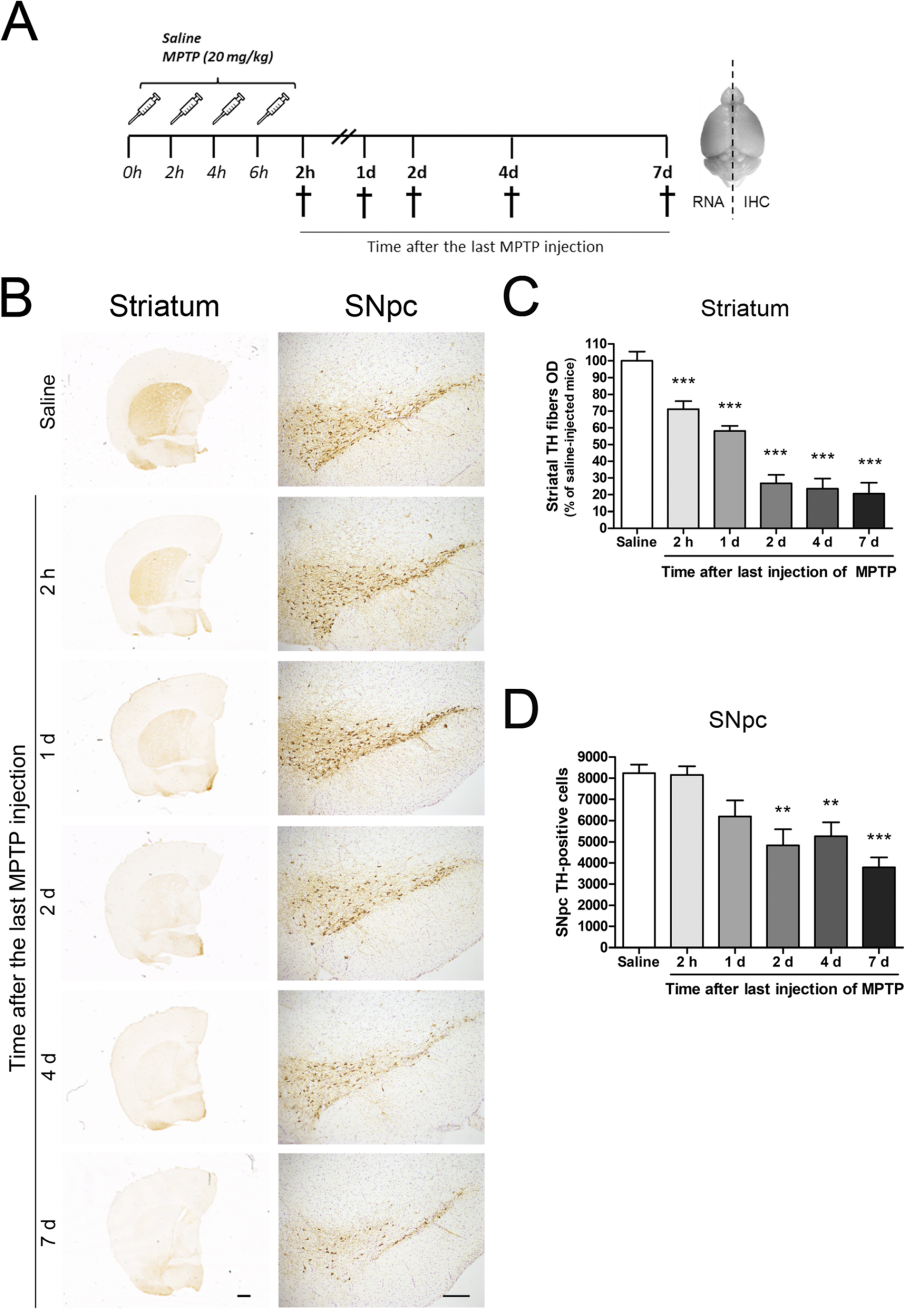
Time course of dopaminergic degeneration in the acute MPTP mouse model. **a** Experimental design. Mice were injected intraperitoneally with saline or MPTP (20 mg/kg) every 2 h to a total of 4 doses in 1 day. Animals were sacrificed at 2 h and at 1, 2, 4, and 7 days (d) after the last injection. Right hemispheres were fixed for immunohistochemistry (IHC) and left hemispheres were processed for gene expression analysis (RNA). **b** TH immunostaining in striatum and substantia nigra pars compacta (SNpc). **c** Optical densitometry of striatal TH-positive dopaminergic fibers. **d** Stereological cell counts of dopaminergic neurons in SNpc. Bars are means + SEM of seven to eight mice per group. **p < 0.01 and ***p < 0.001 vs. saline; one-way ANOVA and Newman-Keuls post hoc test. Scale bars: 500 μm (striatum) and 200 μm (SNpc)

The temporal pattern of dopaminergic neuron damage induced by MPTP administration was assessed at 2 h and at 1, 2, 4 and 7 days after the last MPTP dose (Fig. 1a). We chose 7 days as the last time point because it has previously been reported that the loss of dopaminergic cell bodies in the SNpc is stable at this time [32].

Only one mouse out of a total of 40 (2.5%) died after MPTP administration. TH immunostaining was used to evaluate the extent of dopaminergic neuron damage induced by MPTP in striatum and SNpc. Saline-injected controls showed abundant TH-positive terminals in the striatum and a dense network of cell bodies and fibres in the SNpc (Fig. 1b). MPTP administration led to a gradual reduction of TH immunoreactivity in both brain areas (Fig. 1b). A significant decrease in striatal dopaminergic terminals and SNpc dopaminergic neurons was detected from 2 h after the MPTP challenge and from 2 days afterwards, respectively (Fig. 1c, d). At day 2 after MPTP administration, mice exhibited a dramatic 73% decrease in striatal dopaminergic terminals, which were reduced by 79% at day 7 (Fig. 1c). In the SNpc, MPTP induced a 41% and 54% of dopaminergic neuronal loss at days 2 and 7, respectively (Fig. 1d).

### MPTP-induced neuroinflammation: glial activation and expression of inflammatory markers

**Fig. 2:**
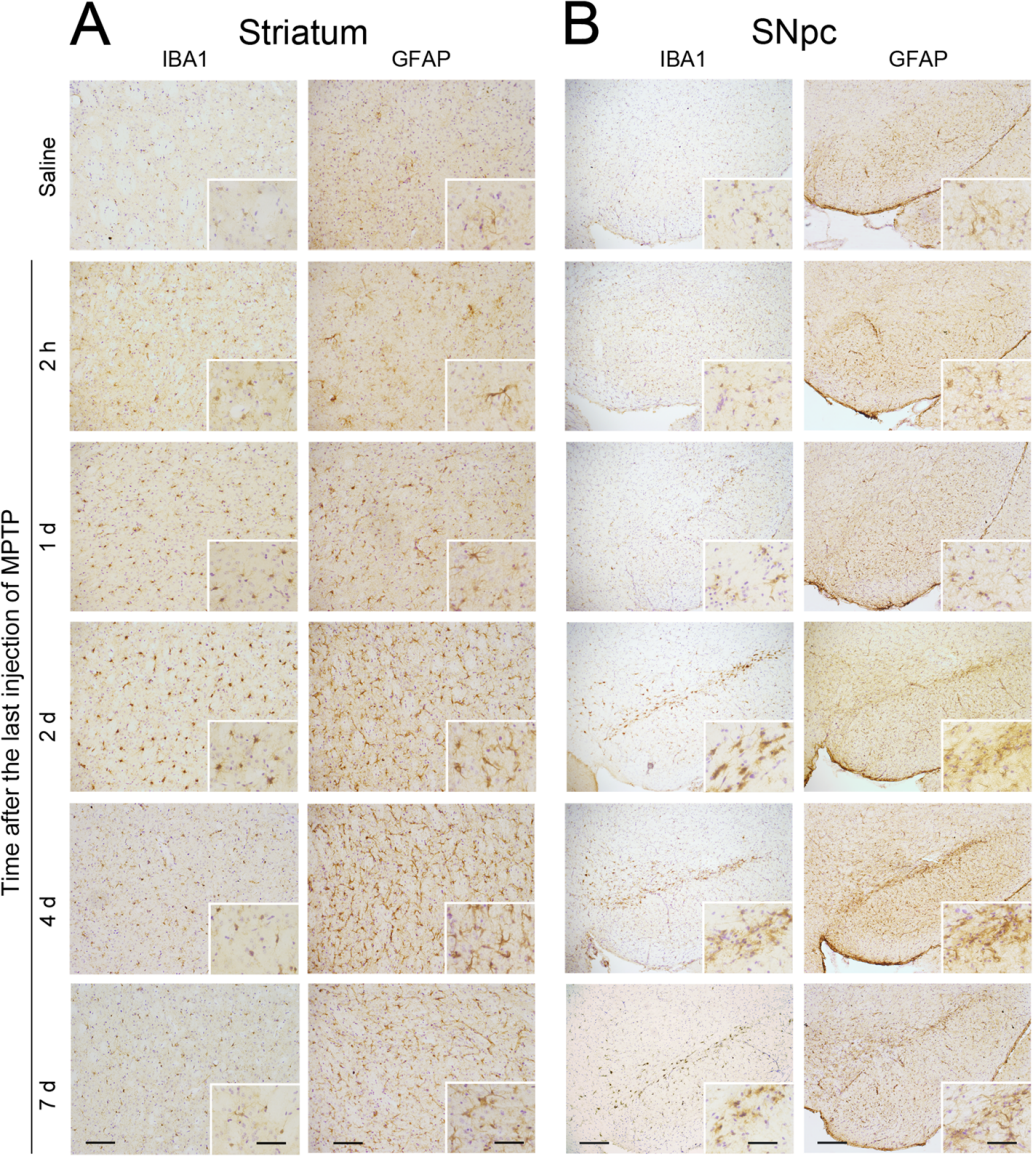
Glial activation associated with MPTP-induced dopaminergic degeneration. **a** Representative photomicrographs of IBA1- and GFAP-immunostained striatum of control mice administered with saline and MPTP-injected mice at the indicated time points after MPTP injections. **b** Representative photomicrographs of IBA1- and GFAP-immunostained substantia nigra pars compacta (SNpc) of control mice administered with saline and MPTP-injected mice at the indicated time points after MPTP injection. Scale bars: 100 μm (striatum), 200 um (SNpc) and 50 μm (insets)

Changes in microglial and astroglial cells in striatum and SN in response to MPTP administration were evaluated by IBA1 and GFAP immunolabelling respectively. An increase in the number of positive cells, with hypertrophied morphology and intensified IBA1 or GFAP staining, was observed throughout the striatum (Table 4, Fig. 2a) and SN, especially in the SNpc (Table 4, Fig. 2b), in MPTP-injected mice. Microglia displayed a reactive phenotype as early as 2 h post-MPTP in the striatum and from day 1 after treatment in the SNpc. In contrast, astroglial reactivity was not evident until day 1 after treatment in both striatum and SNpc. Microglial activation peaked at days 1 to 2 after MPTP injection in the striatum and at days 2 to 4 after MPTP injection in the SNpc. Astroglial activation peaked at days 2 to 4 after MPTP injection in both striatum and SNpc. Both microglia and astroglial activation were attenuated at day 7 after MPTP treatment.

**Table 4:** Qualitative evaluation of the temporal changes in IBA1 and GFAP immunostaining after MPTP treatment

		Time after the last injection of MPTP				
		2 h	1 day	2 days	4 days	7 days
**Striatum**	**IBA1**	+	+++	+++	+	+
	**GFAP**	0	+	+++	+++	++
**SNpc**	**IBA1**	0	++	+++	+++	++
	**GFAP**	0	+	+++	+++	++

Changes in IBA1 and GFAP immunolabelling vs. control mice: 0 = similar to control; + = some cells, ++ = moderate number of cells and +++ = many cells with hypertrophied morphology and intensified IBA1 or GFAP staining

**Fig. 3:**
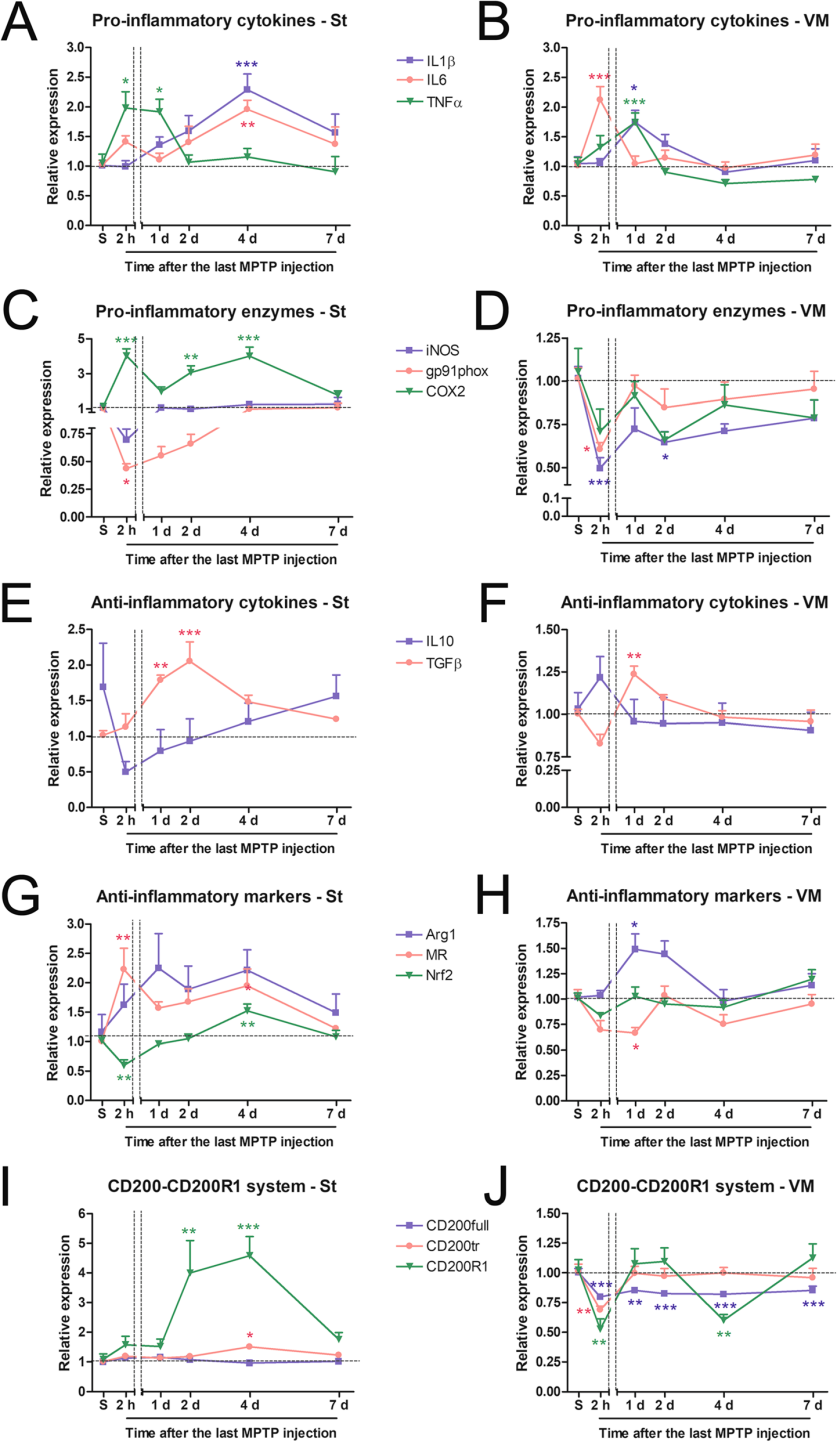
Effect of MPTP administration on the temporal pattern of mRNA expression of molecules involved in the brain inflammatory response. Mice were administered with saline (S) or MPTP and sacrificed at 2 h, and at 1, 2, 4 and 7 days (d) after the last MPTP injection. mRNA expression of the pro-inflammatory cytokines IL1β, IL6 and TNFα (**a**, **b**), the pro-inflammatory enzymes iNOS, COX2 and gp91phox (**c**, **d**), the anti-inflammatory cytokines IL10 and TGFβ (**e**, **f**), the anti-inflammatory markers Arg1, MR and Nrf2 (**g**, **h**) and the components of the CD200-CD200R1 inhibitory system (the ligands CD200full and CD200tr and the receptor CD200R1) (**i**, **j**) in striatum (St, left column) and ventral midbrain (MV, right column) of saline- and MPTP-treated mice. Gapdh and βactin were used as reference genes. Bars are means + SEM of seven to eight mice per experimental group. *p < 0.05, **p < 0.01 and ***p < 0.001 vs. saline control; one-way ANOVA and Newman-Keuls post hoc test

To better characterize the neuroinflammation induced by MPTP treatment, the mRNA levels of pro-inflammatory cytokines (IL1β, IL6 and TNFα) and enzymes (COX2, iNOS and gp91phox) were measured in the striatum and the ventral midbrain of the same saline- and MPTP-injected mice in which TH, IBA1 and GFAP immunostaining were analysed. We also determined the mRNA expression of anti-inflammatory markers, such as the cytokines IL10 and TGFβ, arginase 1 (Arg1), mannose receptor (MR) and the transcription factor nuclear factor erythroid 2-related factor (Nrf2). Pro-inflammatory cytokine mRNA levels in the striatum showed two peaks after MPTP administration: TNFα mRNA increased as early as 2 h afterwards, whereas IL1β and IL6 mRNAs were not significantly increased until day 4 (Fig. 3a). In contrast, ventral midbrain mRNA levels of the three cytokines increased between 2 h and 1 day after MPTP treatment (Fig. 3b). Surprisingly, downregulation of the expression of the pro-inflammatory enzymes gp91phox and iNOS was observed after MPTP administration: gp91phox mRNA levels decreased at 2 h and at day 1 in the two areas analysed, whereas iNOS mRNA levels decreased in the ventral midbrain at 2 h and at day 2 after MPTP administration (Fig. 3c). In contrast, COX2 mRNA levels increased dramatically in the striatum, while no significant changes were observed in the ventral midbrain (Fig. 3d).

Regarding anti-inflammatory markers, TGFβ mRNA levels increased at days 1 and 2 in the striatum (Fig. 3e) and at day 1 in the ventral midbrain (Fig. 3f). IL10 mRNA expression was not significantly modified (Fig. 3e, f). The mRNA level of Arg1 was significantly increased at day 1 in the ventral midbrain (Fig. 3h) and MR mRNA levels increased at 2 h and at day 4 in the striatum (Fig. 3g). In the ventral midbrain, MR mRNA levels decreased at day 1 after MPTP administration (Fig. 3h). Finally, Nrf2 mRNA levels decreased at 2 h but increased at day 4 in the striatum (Fig. 3g). Interestingly, all the MPTP-induced changes observed in the mRNA expression of pro- and anti-inflammatory markers analysed were transient and the mRNA expression returned to basal levels at day 7 following MPTP injection (Fig. 3a–h).

### Changes in the CD200-CD200R1 system after MPTP administration

The temporal pattern of dopaminergic degeneration and neuroinflammation observed after the MPTP challenge was accompanied by changes in CD200 and CD200R1 expression. In the case of the CD200 ligand, we determined the expression of both CD200full mRNA (encoding the full-length molecule) and CD200tr mRNA (encoding a truncated form of the ligand) [8, 9]. In the striatum (Fig. 3i), CD200full mRNA expression was not modified by MPTP administration, but CD200tr mRNA levels showed a significant increase at 4 days after MPTP administration. In striking contrast, CD200R1 mRNA levels were increased dramatically at 2 and 4 days after treatment. In the ventral midbrain (Fig. 3j), a rapid and long-lasting decrease in CD200full was observed, beginning at 2 h after MPTP administration and continuing through 7 days of treatment. CD200tr and CD200R1 mRNAs were transiently decreased 2 h after MPTP injection. Curiously, CD200R1 mRNA also showed a transient decrease at day 4 after MPTP injection while the mRNA levels were equivalent to those of saline-injected animals at days 1, 2 and 7. The expression of CD200full, CD200tr and CD200R1 mRNA returned to basal levels at day 7 after MPTP administration in the two brain areas analysed, with the exception of CD200 mRNA in the ventral midbrain, which remained decreased.

### Modulation of neuronal damage by a CD200R1 agonist

**Fig. 4:**
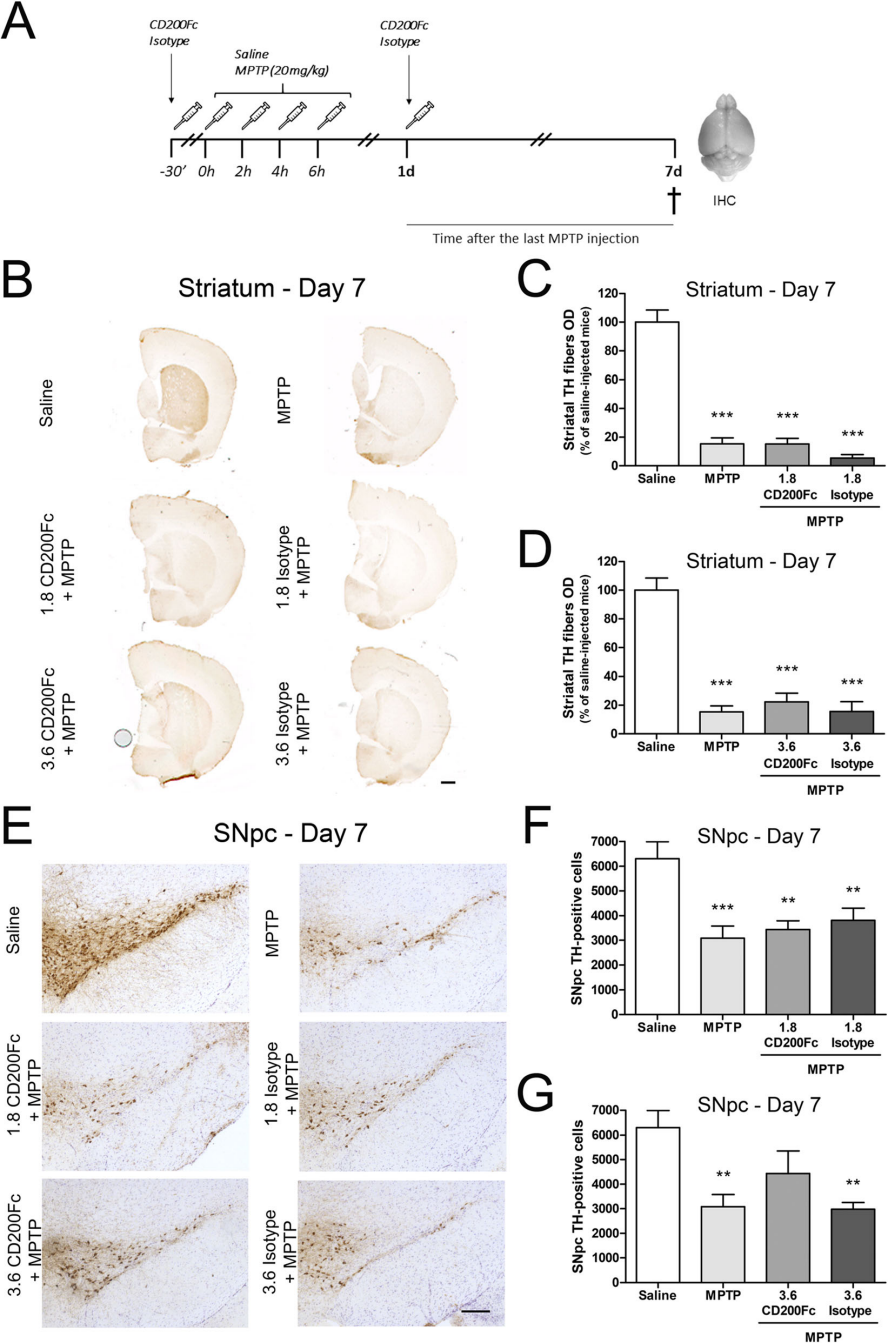
Effect of CD200R1 agonist administration on dopaminergic degeneration in the acute MPTP mouse model. **a** Experimental design. Mice were injected intraperitoneally with saline or MPTP (20 mg/kg) every 2 h to a total of 4 doses in 1 day. In mice treated with the CD200R1 agonist, 1.8 mg/kg or 3.6 mg/kg CD200Fc were intraperitoneally administered twice, 30 min before the first MPTP injection and 24 h after the last one. Isotype administration regimen was the same as for CD200Fc. Mice were sacrificed 7 days (d) following the last MPTP injection and brains were fixed for immunohistochemistry (IHC). **b** TH immunostaining in striatum and optical densitometry of striatal TH-positive dopaminergic fibers of saline, MPTP, CD200Fc + MPTP and isotype+MPTP, considering the lowest dose (1.8 mg/kg) (**c**) and the highest dose (3.6 mg/kg) (**d**) of CD200Fc. **e** TH immunostaining and stereological cell counts of dopaminergic neurons in the substantia nigra pars compacta (SNpc) of saline, MPTP, CD200Fc + MPTP and isotype+MPTP, considering the lowest dose (1.8 mg/kg) (**f**) and the highest dose (3.6 mg/kg) (**g**) of CD200Fc. Bars are means + SEM of five to eight mice per experimental group. **p < 0.01 and ***p < 0.001 vs. saline; one-way ANOVA and Newman-Keuls post hoc test. Scale bars: 500 μm (striatum) and 200 μm (SNpc)

We next studied the effect of CD200-CD200R1 modulation by a CD200R1 agonist, CD200Fc, on nigrostriatal dopaminergic neurodegeneration induced by MPTP. CD200Fc (1.8 mg/kg or 3.6 mg/kg) or the corresponding isotype was administered twice, once before MPTP administration and once after MPTP administration. Mice were sacrificed 7 days after MPTP administration (Fig. 4a).

One mouse from the 1.8 mg/kg CD200Fc + MPTP group (12.5%), three mice from the 1.8 mg/kg isotype+MPTP group (37.5%), two from the 3.6 mg/kg CD200Fc + MPTP group (25%) and one from the 3.6 mg/kg isotype+MPTP group (12.5%) died after MPTP administration.

Optical densitometry of striatal TH-positive terminals showed that MPTP injection produced an 86% depletion of dopaminergic terminals (Fig. 4b–d). The administration of CD200Fc did not prevent this effect. Neither did isotype injection have an effect on the MPTP-induced striatal degeneration. When analysing the neuronal cell bodies of the SNpc, stereological TH-positive cell counts showed that MPTP toxicity induced a significant loss of dopaminergic neurons (51% decrease) (Fig. 4e–g). The administration of the two doses of 1.8 mg/kg of CD200Fc had no effect on MPTP-induced dopaminergic neuronal loss (Fig. 4f). However, this loss was attenuated in the MPTP-injected mice that received the two doses of 3.6 mg/kg CD200Fc (30% decrease) (Fig. 4g). By contrast, a significant decrease in TH-positive neurons was observed in MPTP-treated mice injected with the corresponding isotype (Fig. 4g).

### Dopaminergic damage and microglial activation in MPTP-treated CD200 KO mice

**Fig. 5:**
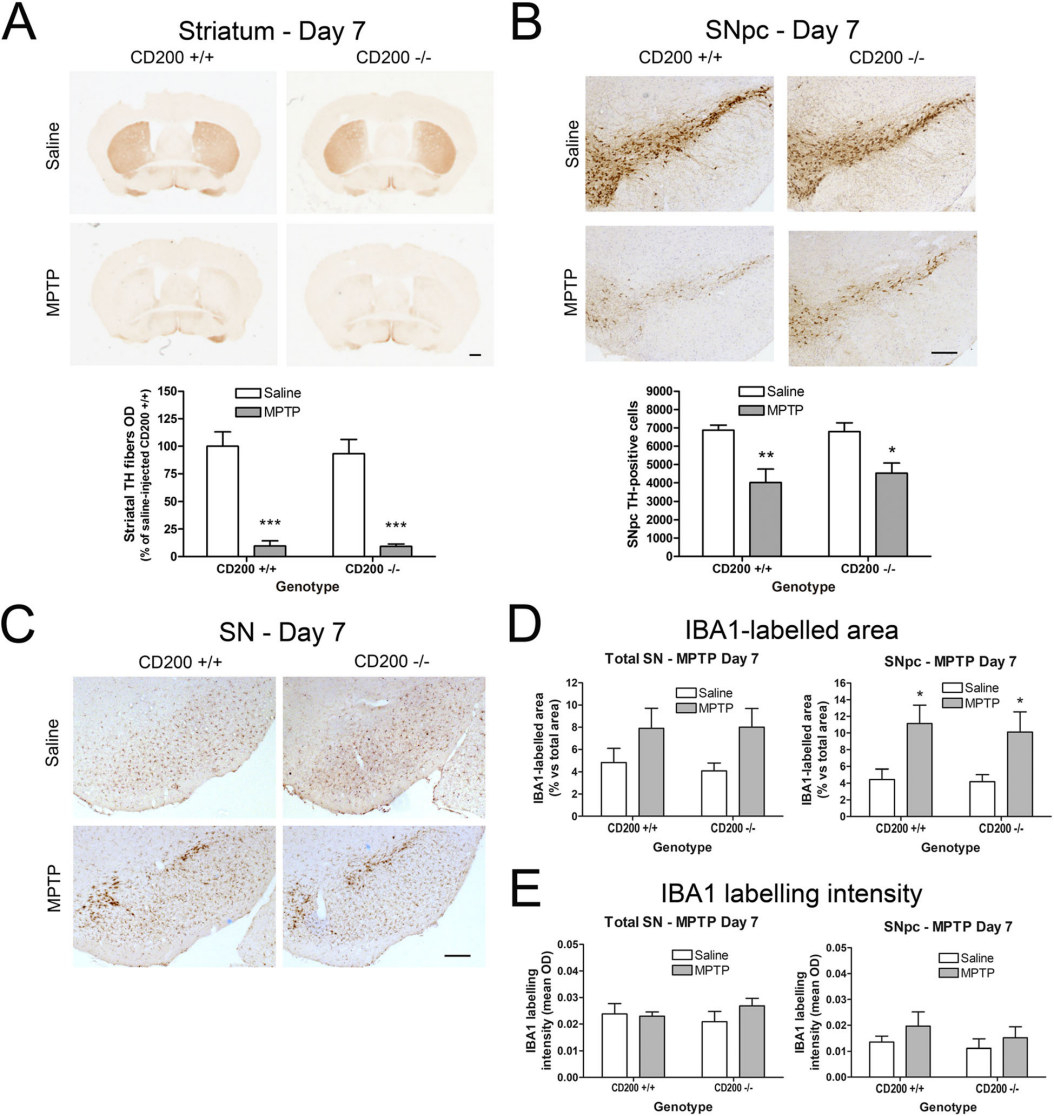
Effect of CD200 deficiency on MPTP-induced dopaminergic neurodegeneration and microglial activation at day 7 after administration. Mice were injected intraperitoneally with saline or MPTP (18 mg/kg) every 2 h to a total of 4 doses in 1 day. Mice were sacrificed 7 days following the last MPTP injection and brains were fixed for immunohistochemistry. **a** TH immunostaining in striatum and optical densitometry of striatal TH-positive dopaminergic fibers of saline- and MPTP-treated CD200 +/+ and CD200 −/− mice. **b** TH immunostaining and stereological cell counts of dopaminergic neurons in the substantia nigra pars compacta (SNpc) of saline- and MPTP-treated CD200 +/+ and CD200 −/− mice. **c** Representative photomicrographs of IBA1-immunostained substantia nigra (SN) of CD200 +/+ and CD200 −/− mice treated with saline or MPTP. Quantification of the IBA1-labelled area (**d**) and the immunolabelling intensity of IBA1-positive cells (**e**) in total SN and SNpc of saline- and MPTP-treated CD200 +/+ and CD200 −/− mice. Bars are means + SEM of five to eight mice per experimental group. *p < 0.05, **p < 0.01 and ***p < 0.001 vs. saline; two-way ANOVA and Bonferroni post hoc test. Scale bars: 500 μm (striatum) and 200 μm (SN and SNpc)

In order to study further the involvement of the CD200-CD200R1 system in the development of dopaminergic neurodegeneration in the acute MPTP model of PD disease, we used CD200-deficient mice. In a first experiment, mice were sacrificed 7 days after MPTP administration (Fig. 5).

In total, seven mice died in the MPTP-injected CD200 +/+ group (58%) and five in the MPTP-injected CD200 −/− group (45.5%). In MPTP-injected CD200 +/+ mice, we observed a significant reduction in the density of dopaminergic fibres in the striatum (90% decrease) (Fig. 5a) and the number of TH-positive cell bodies in the SNpc (42% decrease) (Fig. 5b). In CD200 −/− mice, the striatal TH loss (90% decrease vs. saline CD200 −/−) and the dopaminergic cell death (33% decrease vs. saline CD200 −/−) were similar to those observed in their CD200 +/+ littermates (Fig. 5a, b). We then evaluated whether microglial reactivity was altered in CD200 KO mice looking at IBA1 immunostaining. The IBA1-labelled area in the SN of CD200 −/− mice and their CD200 +/+ littermates showed a tendency to increase at 7 days after MPTP administration (Fig. 5c, d). When analysing SNpc separately, we detected a significant increase in the IBA1-labelled area in both the CD200 −/− and the CD200 +/+ mice treated with MPTP (Fig. 5d). The intensity of the immunolabelling in IBA1-positive cells of the SN was similar in saline- and MPTP-treated mice in both genotypes (Fig. 5e).

**Fig. 6:**
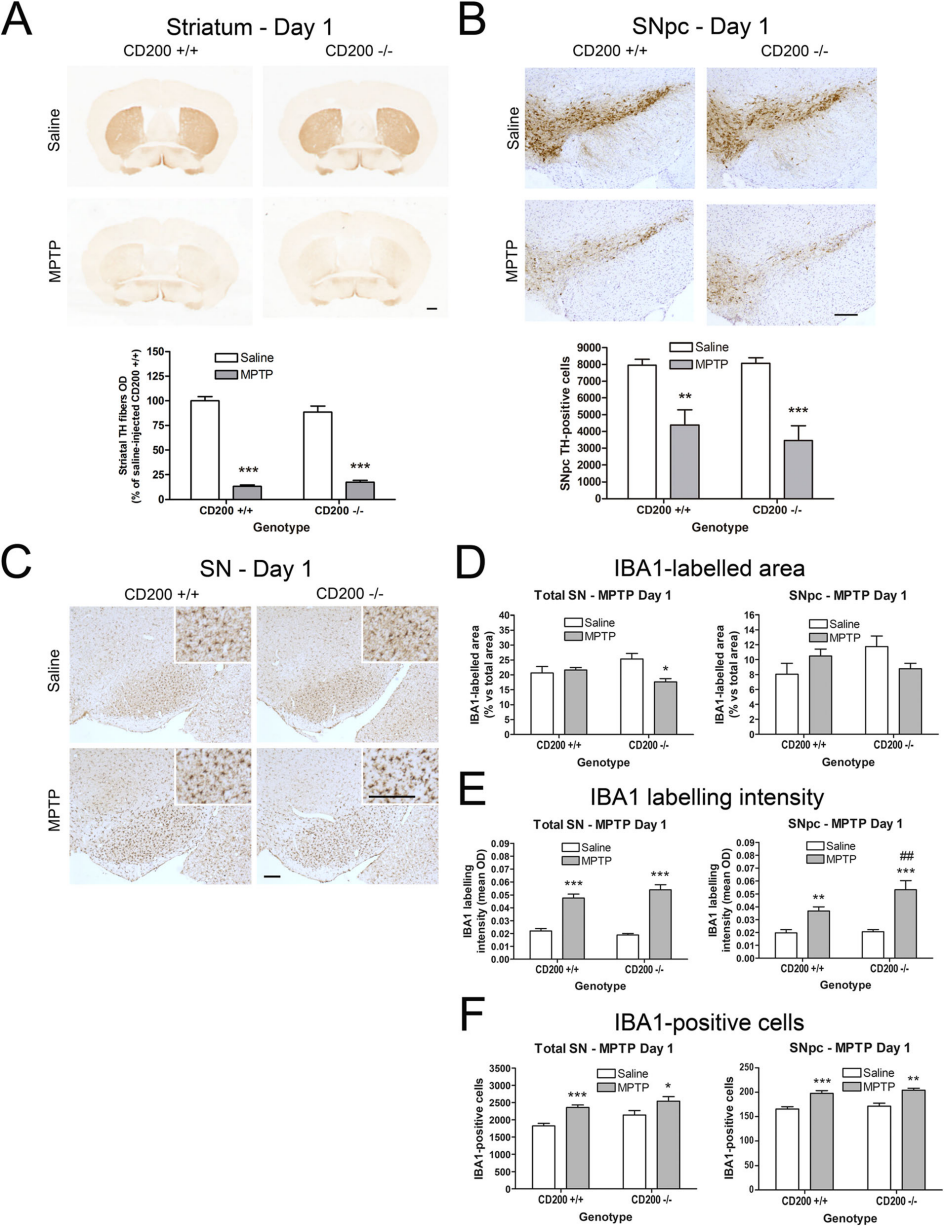
Effect of CD200 deficiency on MPTP-induced dopaminergic neurodegeneration and microglial activation at day 1 after administration. Mice were injected intraperitoneally with saline or MPTP (18 mg/kg) every 2 h to a total of 4 doses in one day. Mice were sacrificed 1 day after the last MPTP injection and brains were fixed for immunohistochemistry. **a** TH immunostaining in striatum and optical densitometry of striatal TH-positive dopaminergic fibers of saline- and MPTP-treated CD200 +/+ and CD200 −/− mice. **b** TH immunostaining and stereological cell counts of dopaminergic neurons in the substantia nigra pars compacta (SNpc) of saline- and MPTP-treated CD200 +/+ and CD200 −/− mice. **c** Representative photomicrographs of IBA1-immunostained substantia nigra (SN) of CD200 +/+ and CD200 −/− mice treated with saline or MPTP. Quantification of the IBA1-labelled area (**d**), the immunolabelling intensity of IBA1-positive cells (**e**) and the total number of IBA1-positive cells in total SN and SNpc of saline- and MPTP-treated CD200 +/+ and CD200 −/− mice. Bars are means + SEM of five to eleven mice per experimental group. *p < 0.05, **p < 0.01 and ***p < 0.001 vs. saline; ##*p* < 0.01 vs. CD200 +/+ MPTP-treated mice; two-way ANOVA and Bonferroni post hoc test. Scale bars: 500 μm (striatum) and 200 μm (SN and SNpc)

To investigate a possible early effect of CD200 deficiency on glial activation and dopaminergic neurodegeneration in the MPTP model, we designed a second experiment in which the CD200 +/+ and the CD200 −/− mice were sacrificed 1 day after the last MPTP injection (Fig. 6). This earlier time point was chosen to check whether MPTP-induced dopaminergic degeneration and microglial activation were accelerated by CD200 deficiency.

Four CD200 −/− mice died after MPTP administration (20% death rate). In CD200 +/+ mice, MPTP administration produced 86.6% depletion of striatal dopaminergic terminals (Fig. 6a) and 44.9% loss of dopaminergic neurons at day 1 post MPTP injection (Fig. 6b). In MPTP-treated CD200 −/− mice, we did not observe further striatal TH-positive fibres loss (80.1% decrease vs. saline CD200 −/−). As regards dopaminergic cells in the SN, no significant differences in the number of TH-positive neurons in SNpc were observed between CD200 −/− mice (57.2% decrease vs. saline CD200 −/−) and CD200 +/+ mice, at 1 day after MPTP injections (Fig. 6b). However, some differences between CD200 +/+ and CD200 −/− mice were observed when looking at microglial activation 1 day after MPTP administration. The IBA1-labelled area was significantly reduced in the total SN (28% decrease) 1 day after MPTP administration in CD200 −/− mice but not in CD200 +/+ mice (Fig. 6c, d). In addition, although the intensity of the immunolabelling in IBA1-positive cells increased similarly in both CD200 +/+ (2.3 times) and CD200 −/− (2.6 times) MPTP-treated mice in total SN (Fig. 6e), a greater increase was observed in the SNpc from CD200 −/− mice (2.6 times) when compared to CD200 +/+ mice (1.9 times) (Fig. 6e). A similar increase in the number of IBA1-positive cells was observed in CD200 +/+ (1.4 times) and CD200 −/− (1.2 times) mice in the SN total and SNpc after MPTP injections (Fig. 6f).

## Discussion

The aim of the present study was to investigate the role of the CD200-CD200R1 system in the development of dopaminergic neuronal death in the acute MPTP model of PD. We observed that MPTP-induced dopaminergic neurodegeneration was accompanied by a transient glial activation in both the striatum and SN, especially in the SNpc. Glial activation and dopaminergic neurodegeneration occurred earlier in the striatum than in the SNpc. In addition, microglial activation occurred before astroglial activation, at least in the striatum. We also observed transient up- and downregulation of inflammatory markers in these brain areas. These effects were accompanied by changes in CD200 and CD200R1 expression, mainly a transient increase in striatal CD200R1 and a sustained decrease in CD200full in the ventral midbrain. The administration of a CD200R1 agonist partially attenuated the MPTP-induced microglial activation and dopaminergic neurodegeneration. Conversely, CD200-deficient mice showed a more activated microglial phenotype in the early stages after MPTP administration.

TH-positive striatal fibres were dramatically reduced to 29% of the control level, as early as 2 h after the last MPTP injection. At this time point, 99% of the TH-positive neurons in the SNpc remained intact. Thus, striatal TH-positive fibres showed a more rapid response to MPTP than did TH-positive cell bodies in the SNpc. Seven days after MPTP injection, when dopaminergic neurodegeneration is stabilized [32], TH-positive striatal fibres were reduced to 21% of the control level, while 46% of the TH-positive neurons still remained. These results suggest that striatal TH-positive fibres are more sensitive to MPTP toxicity than TH-positive neuronal bodies, as previously reported [3, 14, 21, 73].

Previous studies investigated whether glial activation precedes the dopaminergic neurodegeneration after MPTP administration in the acute MPTP mouse model of PD [16, 37, 45]. However, some effects occur so rapidly that it is difficult to determine which occurs first. It is important to point out that we determined the MPTP-induced TH-positive neuron loss and the microglial and astroglial reactivity in the same animals, as well as the mRNA expression profile of pro- and anti-inflammatory markers. In the striatum, we observed that microglial activation and dopaminergic fibre damage, which were detected as early as 2 h after the last MPTP injection, preceded astroglial activation. While Kohutnicka et al. [37] described both microglial and astroglial activation in striatum from day 1, Liberatore et al. [45] reported that microglial activation preceded astroglial activation in the striatum, but without giving details of the time course of events. Liu et al. [48] showed that microglial activation was detected in the striatum just 90 min after MPTP administration, and Suo et al. [71] reported that astroglial activation had not yet been detected in the striatum at day 1 after MPTP administration. Our results corroborate the presence of an earlier microglial than astroglial activation in the striatum which is accompanied by dopaminergic fibre damage. Regarding the SNpc, we detected both microglial and astroglial activation from day 1 after MPTP administration. At this time point, although no significant reduction in the number of TH-positive cells was detected, a tendency to decrease had already been observed. Liberatore et al. [45] described how microglial activation was detected in the ventral midbrain 12 h after MPTP injection, while astroglial activation was only noticeable after 24 h. However, they referred to a previous study [32] to point out that minimal neuronal death occurred at this time. In other studies [16, 37], the earliest time point considered was 1 day after the last MPTP injection, when both microglial activation and neuronal death were present, without addressing the question of whether microglial activation preceded neuronal loss in the SNpc. Our results suggest that both microglial and astroglial activation are already present at initial stages of dopaminergic neuronal death in the SN. We observed that glial activation was transient and that once it reached a peak, IBA1 and GFAP immunostaining started to decrease showing a tendency to return to control levels by day 7 following MPTP administration. This has been described previously [16, 37, 45, 48] and may be due to stabilization of the lesion [32]. By contrast, microglial and astroglial activation is always detected in post-mortem tissue samples of SN in PD patients, suggesting that the neurodegeneration has not stabilized. In addition, imaging studies show enhanced glial activation in PD patients, even at very early disease stages [20, 30]. Recently, it has also been proposed that inflammation at the peripheral level and its posterior transmission to the brain may be one of the initial steps in the development of PD [6, 27]. These observations suggest a role for glial activation in the etiopathogenesis and progress of PD pathology from the preliminary to the final stages.

MPTP-induced glial activation and dopaminergic neuronal damage were accompanied by changes in the mRNA expression of pro- and anti-inflammatory markers in the striatum and ventral midbrain. We detected changes in the expression of inflammatory markers as early as 2 h following the last MPTP injection. Interestingly, the expression of all the markers analysed returned to basal levels at day 7 after MPTP administration, suggesting a resolution of the inflammatory process. In the acute MPTP mouse model, the dynamic profile of pro- and anti-inflammatory markers has mostly been studied in the SN, but little is known about changes in the striatum. In the ventral midbrain, IL1β, IL6 and TNFα mRNA expression were increased in accordance with the mRNA and protein increases described previously using the acute MPTP regimen [10, 11, 34, 36, 71, 81]. We also observed increased IL1β, IL6 and TNFα mRNA expression in the striatum, although with a different temporal pattern than in the ventral midbrain. In the SN in PD patients, increased density of glial cells expressing IL1β and TNFα, as assessed by immunohistochemistry, has been described [29]. Regarding pro-inflammatory enzymes, iNOS and gp91phox mRNA expression was surprisingly decreased in the ventral midbrain and striatum, in contrast to previous observations using the same MPTP regimen [10, 11, 43, 45, 82]. The reason for this divergence is unknown and further studies are required to clarify the temporal pattern of iNOS and gp91phox expression after MPTP administration. COX2 mRNA expression was substantially increased in the striatum and previous studies reported that COX2 mediates microglial activation and dopaminergic cell death in the MPTP mouse model [73, 76]. Interestingly enough, the mRNA levels of anti-inflammatory markers were upregulated (TGFβ, Arg1), downregulated (Nrf2) or showed up- or downregulation in a region-specific manner (MR). Previous reports showed downregulation of anti-inflammatory markers after MPTP administration [66, 86]. In post-mortem and serum human PD samples, alterations both in pro- and anti-inflammatory markers have been also described [19], suggesting a complex pattern of glial activation.

MPTP treatment induced changes in the CD200-CD200R1 system. In the ventral midbrain, a transient decrease in the expression of CD200R1 mRNA was observed as early as 2 h following MPTP injection, suggesting that microglial activation was facilitated. The sustained downregulation of CD200full mRNA expression (mainly expressed by neurons) in the ventral midbrain may reflect early neuronal damage and the final neurodegeneration. A different explanation may apply to CD200tr. CD200tr mRNA expression was transiently decreased at 2 h after MPTP administration in the ventral midbrain, which may be a consequence of early neuronal damage; however, from day 1, CD200tr mRNA expression returned to the basal level. This observation suggests that reactive astrocytes may contribute to CD200tr expression. A different scenario was observed in the striatum, where CD200R1 mRNA was dramatically increased at days 2 and 4 following MPTP administration, suggesting a mechanism aimed at returning microglial activation to basal levels. There are no previous reports on CD200 and CD200R1 mRNA expression in the mouse brain in the MPTP model of PD. However, a gradual decrease in CD200 and CD200R1 protein expression has been previously reported in the whole brain in mice injected with MPTP (subacute regimen), from day 1 to day 7 following MPTP administration [63]. Sung et al. [70] also reported a decrease in the protein expression of CD200 and CD200R1 in the ventral midbrain in the chronic MPTP mouse model, 4 weeks after the last MPTP administration. A long-lasting decrease in CD200R1 and/or CD200 protein expression in the midbrain has also been reported in other in vivo experimental models of PD, such as the LPS model and the adenovirus-induced overexpression of human α-synuclein in SNpc [79] and the 6-OHDA rat model [56]. However, we observed that the mRNA levels of CD200full, CD200tr and CD200R1 returned to basal levels 7 days after MPTP administration, with the exception of CD200full RNA in the ventral midbrain. Curiously, in a recent study [61], we observed a decrease in CD200 and CD200R1 mRNA expression in primary mixed glial cultures treated with MPP+ (MPTP active metabolite), where CD200 is expressed in astrocytes and CD200R1 in microglia. However, MPP+ did not induce a decrease in CD200R1 mRNA expression in primary microglia-enriched cultures [61]. These observations point out the relevance of cell-cell interaction in the effects observed, being the neuronal damage in the in vivo MPTP model an important factor to be taken into account. A decrease in CD200 and or CD200R1 expression has also been detected in the brain of Alzheimer’s disease [77] and multiple sclerosis patients [38, 39] as well as in an experimental model of multiple sclerosis [74]. At present, there are no data on alterations on the cerebral expression of CD200 and CD200R1 in PD, but polymorphisms in the promoter of the CD200R1 gene resulting in a reduced transcriptional activity have been associated with a higher risk of PD [46]. We are currently studying CD200 and CD200R1 expression in post-mortem human brain samples from PD patients.

The CD200-CD200R1 system is an inhibitory mechanism involved in the control of the microglial inflammatory response, and glial activation may play a role in the development of dopaminergic degeneration in the MPTP model. Consequently, we further assessed whether the CD200full- and CD200R1-decreased mRNA levels observed in the ventral midbrain of MPTP-injected mice may play a role in the neuroinflammation and neurodegeneration observed. The administration of a CD200R1 agonist partially attenuated the MPTP-induced dopaminergic neurodegeneration. This attenuation of the MPTP effect was observed at the highest doses of agonist used (3.6 mg/kg CD200Fc) but not at the lowest doses (1.8 mg/kg CD200Fc), suggesting that the effect is concentration dependent. The MPTP-induced loss of striatal dopaminergic nerve terminals was not modified by the CD200R1 agonist, but as exposed above, striatal terminals are more sensitive to MPTP than SNpc dopaminergic cell bodies and it may be more difficult to prevent their loss. However, long-term compensatory dopaminergic sprouting cannot be discarded, as suggested by Bezard et al. [2]. Alternatively, a transient decrease in TH immunoreactivity rather than the loss of nerve terminals may be behind the effect observed, and dopaminergic fibres may require a longer period of time to recover TH immunoreactivity [1]. The recovery of dopaminergic neurotransmission in striatum is critical for normal motor function. The use of higher doses of the CD200R1 agonist or the long-term evaluation of the evolution of TH immunoreactivity in striatum, together with the assessment of motor function, may give additional information on the neuroprotective role of CD200Fc against MPTP-induced dopaminergic neuron damage and derived motor symptoms.

In line with these results, previous studies have shown that the potentiation of the CD200-CD200R1 signalling with a CD200R1 agonist has neuroprotective effects on different experimental models of brain diseases, such as the LPS or the human α-synuclein overexpression models of PD [78, 83], the experimental autoimmune encephalomyelitis model of multiple sclerosis [47] and experimental autoimmune uveoretinitis [13]. In addition, a beneficial role for CD200-CD200R1 signalling has been described in rodent models of brain ischemia using CD200Fc [69, 88] or recombinant CD200 [85]. Several in vitro and in vivo studies showed that the CD200R1 agonist exerts its effect by suppressing the pro-inflammatory microglial activation [15, 24, 25, 33, 47, 51, 79]. In addition, Varnum et al. [75] demonstrated that a CD200R1 agonist stimulated the production of neurotrophic factors in primary microglial cell cultures. By contrast, when CD200R1 is blocked, an exacerbation of microglial activation and dopaminergic neurodegeneration in 6-OHDA-treated rats has been described [87], as well as increased dopaminergic neuron death in the LPS model of PD [83] and a worse outcome in experimental autoimmune encephalomyelitis [55]. Our results show that alterations in the CD200-CD200R1 system may contribute to neurodegeneration in an experimental model of PD that involves dopaminergic neuronal damage, neuroinflammation and mitochondrial dysfunction, and further supports the beneficial effect of a CD200R1 agonist against neuronal damage. This provides further evidence that CD200R1 may be a potential therapeutic target to act against neuroinflammation and its resulting neurotoxicity in PD.

As mentioned above, blocking CD200-CD200R1 signalling has negative consequences on the development of neuronal damage in experimental models of neurological disorders [55, 83, 87]. Although there are few studies using CD200 or CD200R1 KO mice, especially in the context of the CNS, an accelerated microglial response or a worse outcome of the pathology in CD200 KO mice has also been described in experimental models of neuronal damage such as experimental autoimmune uveoretinitis, encephalitis, facial nerve transection or experimental autoimmune encephalomyelitis (EAE) [5, 17, 26] and in stroke in CD200R1 mice [65]. In our acute MPTP model of PD, the CD200-deficient mice presented the same degree of MPTP-induced microglial activation and dopaminergic neurodegeneration as their wild-type littermates 7 days after the last MPTP injection, when the lesion has stabilized [32]. Several authors suggest that inhibition of CD200-CD200R1 signalling in peripheral cells contributes to the development of the inflammatory response in the damaged brain [65, 83], which probably plays a more relevant role in EAE and ischemia, where cellular infiltrates are critically involved in the pathological outcome, than in the MPTP model. Surprisingly, a similar degree of dopaminergic neurodegeneration was observed as early on as day 1, suggesting that the mice used in the present experiment (CD200 +/+ and CD200 −/− colonies) presented a higher sensitivity to MPTP toxicity than the mice we had used in the previous experiments (C57Bl/6N from Charles River). This observation was corroborated by the higher mortality rate after MPTP injections in the former, which made us to reduce the MPTP dosage in the experiments with the KO mice. However, microglial cells presented a more activated phenotype in MPTP-injected CD200-deficient mice than in the wild-type littermates 1 day after the last MPTP injection, including a decreased IBA1-labelled area, larger cell bodies and shortened projections, as well as increased intensity of the IBA1 immunolabelling in SNpc, without an increase in the number of microglial cells. Nevertheless, this increased microglial activation was not accompanied by an enhanced MPTP-induced dopaminergic neurodegeneration. A possible explanation is that the MPTP dose administered was too high, due to the higher sensitivity of the mice to the MPTP toxicity mentioned above, to observe any difference in dopaminergic neurodegeneration between CD200-deficient mice and their wild-type littermates. Even in the wild-type group, at day 1 post MPTP injections, there was already a 90% decrease in TH-positive fibres in the striatum and a 42% decrease in TH-positive cells in the SNpc, values that we did not obtain until 7 days after MPTP administration in the time course experiment. These differences could be due to the different genetic backgrounds of the two substrains used in the experiments: 100% C57BL/6N in the case of Charles River mice and 66% C57BL/6N + 33% C57BL/6J in the case of the CD200 KO colony. In fact, previous studies have shown that different substrains have differing sensitivity to MPTP [22, 23, 31]. It is possible that the use of lower doses of MPTP would help us to appreciate differences between wild-type and CD200 KO mice. These results point to the importance of using mice with identical genetic backgrounds when comparing results from experiments with wild-type control mice and KO mice, and of carefully reporting the genetic background in mouse MPTP studies. The lack of effect of CD200 deficiency on dopaminergic damage in our MPTP model could also be due to the fact that the absence of CD200 from the very beginning in constitutive CD200 KO mice may activate compensatory mechanisms that may mask the real effect of the absence of CD200 occurring in a pathological condition. In this sense, the use of conditional KO mice, where time-specific deletion of CD200 or CD200R1 gene expression could be induced, would offer additional advantages.

Previous reports, using CD200-deficient mice from the same source as ours, have shown that in the spleen CD11b + cells doubled in size and macrophage and dendritic cells showed increased activation, whereas lymph nodes were slightly enlarged with expanded and activated macrophages [26]. Furthermore, microglia of CD200 −/− mice spontaneously exhibited many features of activation (less ramified, shorter glial processes, disordered arrangement, increased CD11b and CD45 expression) and formed aggregates, especially in the spinal cord [26]. Hoek and collaborators reported no evidence of T cell deregulation in CD200-deficient mice [26]. Although we observed that microglial cells were apparently more activated in MPTP-treated CD200 KO mice 1 day after the injection, no differences were observed between microglial cells of the saline-treated WT and CD200 KO mice. Curiously, Wang et al. [78] reported a selective decrease in the number of dopaminergic neurons in the SN of 5-month-old CD200 KO mice, but not in 3-month-old mice, suggesting that a spontaneous dopaminergic neurodegeneration occurs in CD200 KO mice. However, the CD200 KO mice were different from those used in the present study and the mice we used were always less than 5 months old.

## Conclusions

Taken together, our results show that the CD200-CD200R1 system is altered in the MPTP experimental model of PD, suggesting that the absence of CD200 leads to a more activated microglial phenotype and that CD200R1 activation may attenuate the progression of neuronal damage in PD. Therapeutic interventions aimed at preventing microglial activation through potentiation of CD200-CD200R1 signalling may be a useful novel approach in PD treatment.

## Data Availability

All data generated or analysed during this study are included in this published article.

## Abbreviations

ANOVA: Analysis of variance
Arg1: arginase
CD200full: full-lenght CD200
CD200tr: truncated CD200
CD200R1: CD200 R1 receptor
CD200Fc: CD200-Fc fusion protein, a CD200R1 agonist
COX2: Cyclooxygenase 2
EAE: Experimental autoimmune encephalomyelitis
Gapdh: glyceraldehyde-3-phosphate dehydrogenase
GFAP: Glial fibrillary acidic protein
gp91phox: catalytic subunit of NADPH oxidase
IBA1: Ionized calcium binding adaptor molecule 1
IHC: immunohistochemistry
IL1: Interleukin
i.p.: intraperitoneal
iNOS: inducible nitric oxide synthase
KO: knockout
MPTP: 1-methyl-4-phenyl-1,2,3,6-tetrahydropyridine
MR: mannose receptor
Nrf2: nuclear factor erythroid 2-related factor 2
PBS: Phosphate-buffered saline
PD: Parkinson’s disease
qRT-PCR: quantitative real-time polymerase chain reaction
SN: Substantia nigra
SNpc: Substantia nigra pars compacta
TGFβ: Transforming growth factor beta
TH: Tyrosine hydroxylase
TNFα: Tumor necrosis factor alpha
TBS: Tris-buffered saline
